# Supplementary material for: A Statistical Framework to Predict Functional Non-Coding Regions in the Human Genome Through Integrated Analysis of Annotation Data
Source: Sci Rep. 2015 Jun 30;5:10576. doi: 10.1038/srep10576 (PMC4444969; doi:10.1038/srep10576)
Supplement: Supplementary Information [file srep10576-s1.pdf]

A Statistical Framework to Predict Functional Non-Coding Regions in the Human Genome Through Integrated Analysis of Annotation Data

Qiongshi Lu[1], Yiming Hu[1], Jiehuan Sun[1], Yuwei Cheng[2], Kei-Hoi Cheung[2,3,4,5], Hongyu Zhao[1,2,5*]

[1]Department of Biostatistics, Yale School of Public Health, New Haven, CT, USA

[2]Program of Computational Biology and Bioinformatics, Yale University, New Haven, CT, USA

[3]Yale Center for Medical Informatics, Yale School of Medicine, New Haven, CT, USA

[4]Department of Emergency Medicine, Yale School of Medicine, New Haven, CT, USA

[5]VA Connecticut Healthcare System, West Haven, CT, USA

*Correspondence:

Dr. Hongyu Zhao
Department of Biostatistics
Yale School of Public Health
60 College Street,
New Haven, CT, 06511, USA
hongyu.zhao@yale.edu

Supplementary Materials

Supplementary Table 1. The 22 annotations used in the model

Notation	Annotation	Category
A_1	GERP	Conservation Measure
A_2	PhyloP	
A_3	DNase I	Open Chromatin
A_4	FAIRE	
A_5	H3k4me1	Histone Modification
A_6	H3k4me2	
A_7	H3k4me3	
A_8	H3k9ac	
A_9	H3k27ac	
A_{10}	H3k27me3	
A_{11}	H3k36me3	
A_{12}	H4k20me1	
A_{13}	CEBPB	TFBS
A_{14}	CTCF	
A_{15}	EP300	
A_{16}	FOS	
A_{17}	GATA2	
A_{18}	JUND	
A_{19}	MAX	
A_{20}	MYC	
A_{21}	POLR2A	
A_{22}	RAD21	

Supplementary Table 2. Predicted functional proportion for each chromosome using 0.5 as the cutoff

Chromosome	Proportion	Chromosome	Proportion
1	0.332	13	0.365
2	0.331	14	0.344
3	0.356	15	0.392
4	0.293	16	0.338
5	0.374	17	0.334
6	0.316	18	0.362
7	0.321	19	0.313
8	0.328	20	0.340
9	0.388	21	0.331
10	0.337	22	0.383
11	0.322	X	0.281
12	0.307	Y	0.193
**Overall**	**0.333**		

Supplementary Table 3. Online sources for the 22 annotations

Annotation	Website
GERP	http://mendel.stanford.edu/SidowLab/downloads/gerp/
PhyloP	http://hgdownload.soe.ucsc.edu/goldenPath/hg19/phyloP46way/
DNase I	http://hgdownload.cse.ucsc.edu/goldenPath/hg19/encodeDCC/wgEncodeRegDnaseClustered/
FAIRE	http://ftp.ebi.ac.uk/pub/databases/ensembl/encode/integration_data_jan2011/byDataType/openchrom/jan2011/faire_fseq_peaks/
H3k4me1	http://genome.ucsc.edu/cgi-bin/hgFileUi?db=hg19&g=wgEncodeBroadHistone/
H3k4me2	
H3k4me3	
H3k9ac	
H3k27ac	
H3k27me3	
H3k36me3	
H4k20me1	
CEBPB	http://hgdownload.cse.ucsc.edu/goldenPath/hg19/encodeDCC/wgEncodeRegTfbsClustered/
CTCF	
EP300	
FOS	
GATA2	
JUND	
MAX	
MYC	
POLR2A	
RAD21	

Supplementary Table 4. 16 Cell lines used to cluster the histone peak signal

Cell Line
A549
Dnd41
Gm12878
H1hesc
Helas3
Hepg2
Hmec
Hsmm
Hsmmt
Huvec
K562
Monocd14ro1746
Nha
Nhdfad
Nhek
Nhlf

Supplementary Table 5. Estimates of all 49 parameters and the odds ratios for binary annotations

Parameter	Estimate (Z=1)	Parameter	Estimate (Z=0)	Odds Ratio
π	0.4274	$1-\pi$	0.5726	-
μ_{11}	-0.1414	μ_{10}	-0.0608	-
μ_{21}	0.1249	μ_{20}	0.0742	-
σ_{11}	2.5274	σ_{10}	1.6262	-
σ_{21}	1.0765	σ_{20}	0.6623	-
p_{31}	0.3145	p_{30}	0.0610	7.06
p_{41}	0.3061	p_{40}	0.0941	4.25
p_{51}	0.9535	p_{50}	0.3016	47.48
p_{61}	0.7633	p_{60}	0.0943	30.97
p_{71}	0.6562	p_{70}	0.0634	28.20
p_{81}	0.7994	p_{80}	0.1418	24.12
p_{91}	0.8712	p_{90}	0.2085	25.68
$p_{10,1}$	0.7983	$p_{10,0}$	0.7248	1.50
$p_{11,1}$	0.8287	$p_{11,0}$	0.3764	8.01
$p_{12,1}$	0.9246	$p_{12,0}$	0.6360	7.02
$p_{13,1}$	0.0360	$p_{13,0}$	0.0041	9.07
$p_{14,1}$	0.0509	$p_{14,0}$	0.0060	8.88
$p_{15,1}$	0.0480	$p_{15,0}$	0.0017	29.61
$p_{16,1}$	0.0359	$p_{16,0}$	0.0018	20.65
$p_{17,1}$	0.0276	$p_{17,0}$	0.0026	10.89
$p_{18,1}$	0.0312	$p_{18,0}$	0.0015	21.44
$p_{19,1}$	0.0371	$p_{19,0}$	0.0003	128.39
$p_{20,1}$	0.0383	$p_{20,0}$	0.0007	56.85
$p_{21,1}$	0.1002	$p_{21,0}$	0.0023	48.31
$p_{22,1}$	0.0278	$p_{22,0}$	0.0020	14.27

Supplementary Table 6. Estimates of all 49 parameters with the missing conservation values replaced by 0

Parameter	Estimate (Z=1)	Parameter	Estimate (Z=0)
π	0.4305	$1-\pi$	0.5695
μ_{11}	-0.1676	μ_{10}	-0.0377
μ_{21}	0.1142	μ_{20}	0.0791
σ_{11}	2.5549	σ_{10}	1.5445
σ_{21}	1.0886	σ_{20}	0.6269
p_{31}	0.3051	p_{30}	0.0621
p_{41}	0.2976	p_{40}	0.0949
p_{51}	0.9493	p_{50}	0.3646
p_{61}	0.7789	p_{60}	0.1233
p_{71}	0.6659	p_{70}	0.0765
p_{81}	0.8024	p_{80}	0.1688
p_{91}	0.8552	p_{90}	0.2286
$p_{10,1}$	0.8442	$p_{10,0}$	0.7696
$p_{11,1}$	0.8493	$p_{11,0}$	0.4570
$p_{12,1}$	0.9397	$p_{12,0}$	0.7115
$p_{13,1}$	0.0355	$p_{13,0}$	0.0038
$p_{14,1}$	0.0500	$p_{14,0}$	0.0057
$p_{15,1}$	0.0468	$p_{15,0}$	0.0018
$p_{16,1}$	0.0348	$p_{16,0}$	0.0019
$p_{17,1}$	0.0272	$p_{17,0}$	0.0024
$p_{18,1}$	0.0305	$p_{18,0}$	0.0015
$p_{19,1}$	0.0362	$p_{19,0}$	0.0004
$p_{20,1}$	0.0377	$p_{20,0}$	0.0005
$p_{21,1}$	0.0974	$p_{21,0}$	0.0028
$p_{22,1}$	0.0273	$p_{22,0}$	0.0019

Supplementary Table 7. Estimates of all 49 parameters when an extra sample of randomly chosen 2,000,000 positions on chromosome 1 was added into the original dataset

Parameter	Estimate (Z=1)	Parameter	Estimate (Z=0)
π	0.4332	$1-\pi$	0.5668
μ_{11}	-0.1648	μ_{10}	-0.0334
μ_{21}	0.1162	μ_{20}	0.0805
σ_{11}	2.5571	σ_{10}	1.5362
σ_{21}	1.0876	σ_{20}	0.6247
p_{31}	0.3058	p_{30}	0.0593
p_{41}	0.2937	p_{40}	0.0935
p_{51}	0.9455	p_{50}	0.3382
p_{61}	0.7673	p_{60}	0.1094
p_{71}	0.6456	p_{70}	0.0642
p_{81}	0.7880	p_{80}	0.1476
p_{91}	0.8516	p_{90}	0.2276
$p_{10,1}$	0.8388	$p_{10,0}$	0.7393
$p_{11,1}$	0.8371	$p_{11,0}$	0.4247
$p_{12,1}$	0.9392	$p_{12,0}$	0.6928
$p_{13,1}$	0.0353	$p_{13,0}$	0.0037
$p_{14,1}$	0.0497	$p_{14,0}$	0.0057
$p_{15,1}$	0.0464	$p_{15,0}$	0.0017
$p_{16,1}$	0.0351	$p_{16,0}$	0.0019
$p_{17,1}$	0.0276	$p_{17,0}$	0.0024
$p_{18,1}$	0.0307	$p_{18,0}$	0.0014
$p_{19,1}$	0.0359	$p_{19,0}$	0.0004
$p_{20,1}$	0.0379	$p_{20,0}$	0.0005
$p_{21,1}$	0.0957	$p_{21,0}$	0.0025
$p_{22,1}$	0.0270	$p_{22,0}$	0.0018

Supplementary Table 8. Estimates of all 49 parameters when an extra sample of randomly chosen 6,000,000 positions on chromosome 1 was added into the original dataset

Parameter	Estimate (Z=1)	Parameter	Estimate (Z=0)
π	0.4393	$1-\pi$	0.5607
μ_{11}	-0.1747	μ_{10}	-0.0126
μ_{21}	0.1135	μ_{20}	0.0824
σ_{11}	2.5745	σ_{10}	1.4384
σ_{21}	1.0908	σ_{20}	0.5882
p_{31}	0.3015	p_{30}	0.0523
p_{41}	0.2838	p_{40}	0.0886
p_{51}	0.9317	p_{50}	0.2969
p_{61}	0.7388	p_{60}	0.0884
p_{71}	0.6056	p_{70}	0.0477
p_{81}	0.7566	p_{80}	0.1150
p_{91}	0.8387	p_{90}	0.2214
$p_{10,1}$	0.8298	$p_{10,0}$	0.6918
$p_{11,1}$	0.8120	$p_{11,0}$	0.3750
$p_{12,1}$	0.9362	$p_{12,0}$	0.6575
$p_{13,1}$	0.0342	$p_{13,0}$	0.0034
$p_{14,1}$	0.0482	$p_{14,0}$	0.0051
$p_{15,1}$	0.0448	$p_{15,0}$	0.0015
$p_{16,1}$	0.0347	$p_{16,0}$	0.0018
$p_{17,1}$	0.0277	$p_{17,0}$	0.0021
$p_{18,1}$	0.0303	$p_{18,0}$	0.0012
$p_{19,1}$	0.0346	$p_{19,0}$	0.0003
$p_{20,1}$	0.0373	$p_{20,0}$	0.0004
$p_{21,1}$	0.0911	$p_{21,0}$	0.0019
$p_{22,1}$	0.0259	$p_{22,0}$	0.0016

Supplementary Table 9. Estimates of parameters when GERP, DNase I, H3k4me2 and H4k20me1, and CEBPB and MAX are dropped from the model, respectively

Param.	**Estimate (Z=1)** Without GERP	**Estimate (Z=1)** Without DNaseI	**Estimate (Z=1)** Without H3k4me2 H4k20me1	**Estimate (Z=1)** Without CEBPB MAX	Param.	**Estimate (Z=0)** Without GERP	**Estimate (Z=0)** Without DNaseI	**Estimate (Z=0)** Without H3k4me2 H4k20me1	**Estimate (Z=0)** Without CEBPB MAX
π	0.4201	0.4361	0.4417	0.4375	$1-\pi$	0.5799	0.5639	0.5583	0.5625
μ_{11}	-	-0.1671	-0.2468	-0.1696	μ_{10}	-	-0.0368	0.0277	-0.0344
μ_{21}	0.1362	0.1131	0.0902	0.1126	μ_{20}	0.0637	0.0796	0.0974	0.0799
σ_{11}	-	2.5469	2.6781	2.5510	σ_{10}	-	1.5412	1.3288	1.5324
σ_{21}	1.0328	1.0855	1.1355	1.0861	σ_{20}	0.7009	0.6249	0.5433	0.6225
p_{31}	0.3069	-	0.3039	0.3015	p_{30}	0.0651	-	0.0582	0.0618
p_{41}	0.3001	0.2933	0.2961	0.2948	p_{40}	0.0967	0.0962	0.0921	0.0946
p_{51}	0.9653	0.9484	0.9175	0.9467	p_{50}	0.3633	0.3594	0.3779	0.3593
p_{61}	0.7970	0.7736	-	0.7724	p_{60}	0.1219	0.1208	-	0.1202
p_{71}	0.6817	0.6623	0.6425	0.6594	p_{70}	0.0755	0.0733	0.0831	0.0742
p_{81}	0.8181	0.7998	0.7802	0.7978	p_{80}	0.1687	0.1645	0.1736	0.1645
p_{91}	0.8725	0.8562	0.8357	0.8517	p_{90}	0.2272	0.2215	0.2314	0.2235
$p_{10,1}$	0.8438	0.8431	0.8423	0.8441	$p_{10,0}$	0.7712	0.7697	0.7696	0.7688
$p_{11,1}$	0.8574	0.8513	0.8390	0.8476	$p_{11,0}$	0.4581	0.4515	0.4573	0.4534
$p_{12,1}$	0.9417	0.9398	-	0.9394	$p_{12,0}$	0.7141	0.7091	-	0.7089
$p_{13,1}$	0.0361	0.0347	0.0352	-	$p_{13,0}$	0.0039	0.0041	0.0034	-
$p_{14,1}$	0.0505	0.0483	0.0501	0.0492	$p_{14,0}$	0.0061	0.0066	0.0047	0.0058
$p_{15,1}$	0.0477	0.0458	0.0460	0.0460	$p_{15,0}$	0.0020	0.0021	0.0015	0.0018
$p_{16,1}$	0.0355	0.0338	0.0346	0.0342	$p_{16,0}$	0.0020	0.0024	0.0014	0.0020
$p_{17,1}$	0.0274	0.0266	0.0272	0.0268	$p_{17,0}$	0.0027	0.0026	0.0019	0.0023
$p_{18,1}$	0.0311	0.0300	0.0302	0.0300	$p_{18,0}$	0.0015	0.0016	0.0011	0.0015
$p_{19,1}$	0.0370	0.0357	0.0355	-	$p_{19,0}$	0.0004	0.0005	0.0003	-
$p_{20,1}$	0.0385	0.0370	0.0369	0.0370	$p_{20,0}$	0.0006	0.0006	0.0004	0.0006
$p_{21,1}$	0.0994	0.0958	0.0958	0.0960	$p_{21,0}$	0.0030	0.0031	0.0022	0.0028
$p_{22,1}$	0.0276	0.0263	0.0275	0.0269	$p_{22,0}$	0.0021	0.0024	0.0012	0.0019

Supplementary Figure 1. Histograms of prediction score in 24 chromosomes.

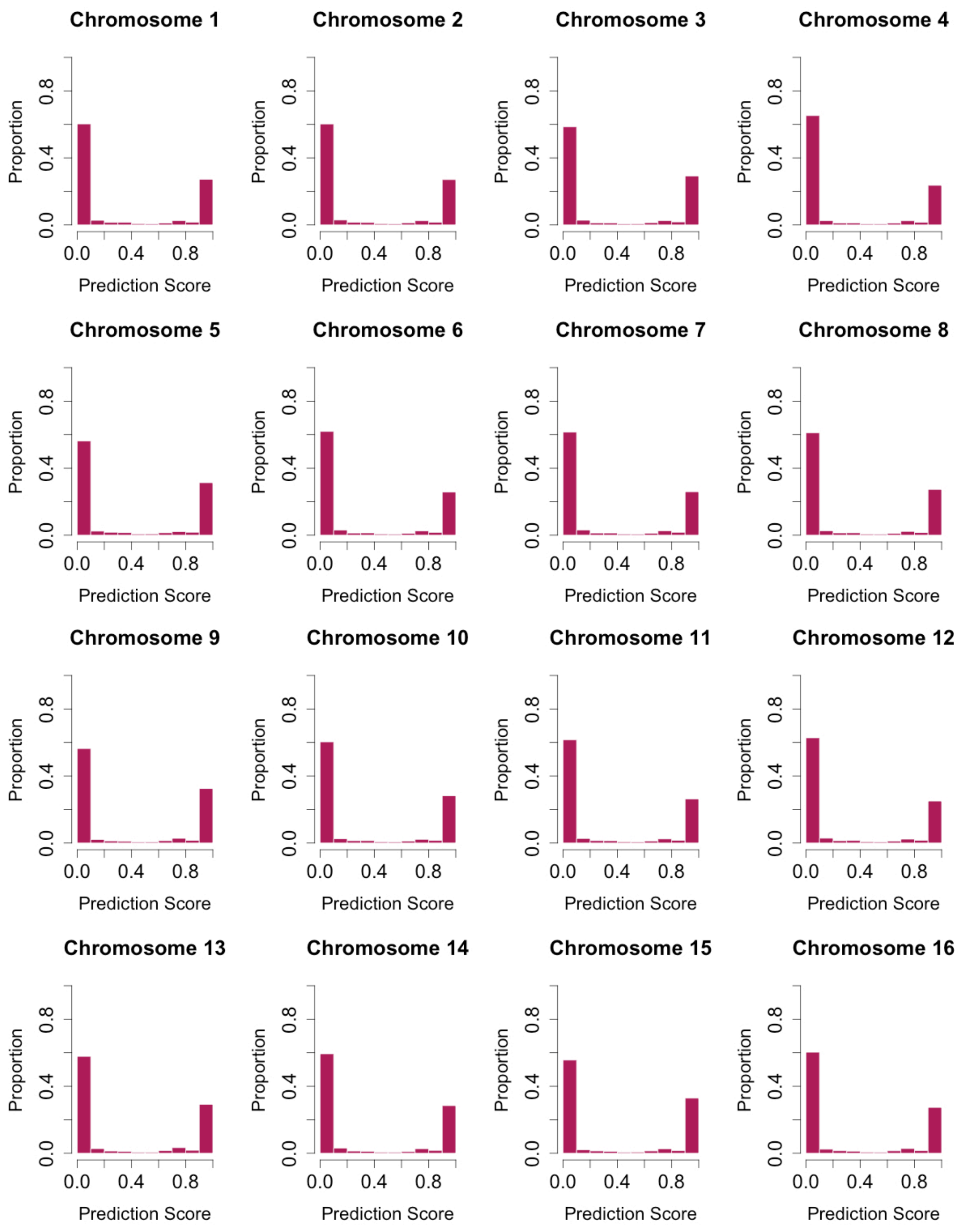

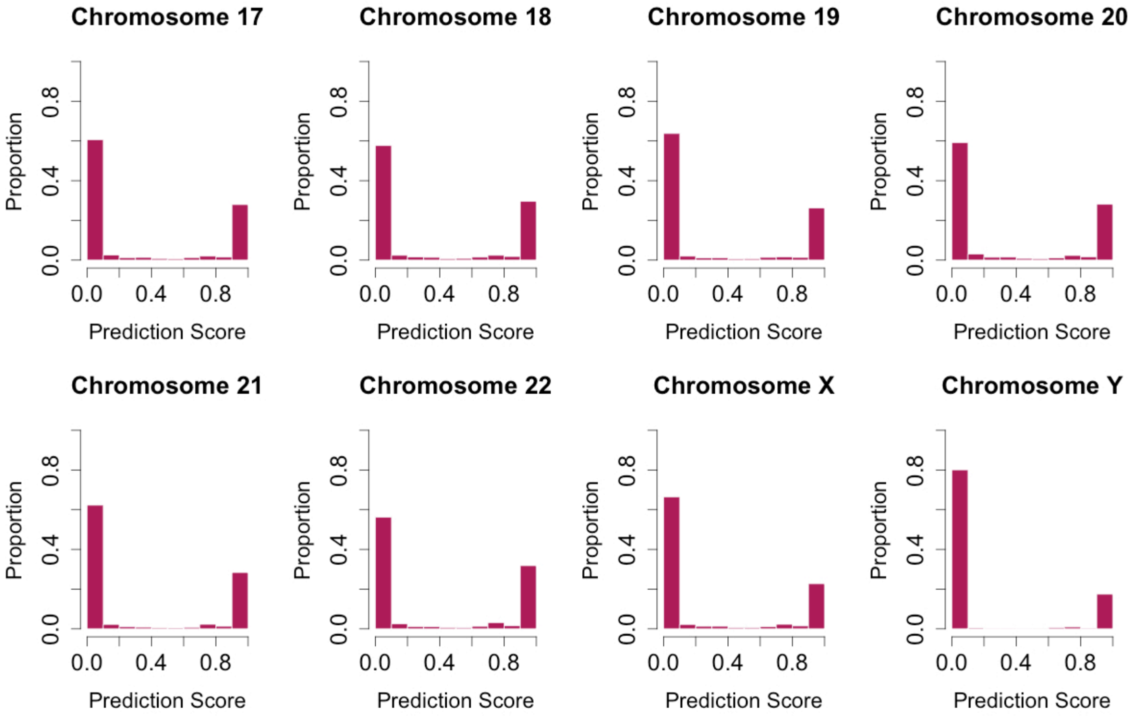
Chromosome 17
Chromosome 18
Chromosome 19
Chromosome 20
Chromosome 21
Chromosome 22
Chromosome X
Chromosome Y
Proportion
0.0
0.4
0.8
Prediction Score
